# Clear scalp, clear mind: Examining the beneficial impact of dandruff reduction on physical, emotional and social wellbeing

**DOI:** 10.1111/ics.13041

**Published:** 2025-01-21

**Authors:** A. Newton‐Fenner, H. Roberts, M. Scott, T. Jones, L. Collins, A. Godbehere, T. Giesbrecht

**Affiliations:** ^1^ Unilever Research and Development Port Sunlight Laboratory Wirral UK; ^2^ Department of Psychological Sciences, Institute of Population Health University of Liverpool Liverpool UK

**Keywords:** dandruff, emotional wellbeing, mediation analysis, scalpdex, self‐esteem

## Abstract

**Objective:**

Dandruff is a common scalp condition characterized by the chronic shedding of large clumps of epidermal cells. Alongside negative physical symptoms, dandruff is thought to have a detrimental impact on individuals' mental health. The aim of this study was to determine whether the clinical benefits of using an anti‐dandruff shampoo (ADS) will result in improved psychological wellbeing in individuals suffering from dandruff. Further, this study aimed to investigate whether this improvement would manifest in changes in self‐esteem and confidence behaviours.

**Methods:**

A 4‐week dandruff reduction intervention was implemented using a Piroctone Olamine ADS formulation. The control group continued to use their non‐ADS. Self‐evaluation metrics, including the Scalpdex, State Self‐Esteem Scale and self‐perceived hair and scalp characteristics questionnaires, were employed to measure changes in self‐perceptions of hair and scalp health and psychological wellbeing. Following this analysis, the dataset was then combined with unpublished internal data of a similar intervention study using a Zinc Pyrithione ADS formulation. A mediation analysis was conducted on the combined data to examine the relationship between dandruff symptoms, emotions, and overall behavioural functioning.

**Results:**

First, the ADS effectively reduced the clinical symptoms of dandruff. Second, Scalpdex scores indicated that the use of ADS, compared to non‐ADS, lessened the adverse effects of dandruff on symptom perception and emotional distress. Third, the impact of dandruff on behavioural functioning diminished over time, and measures of confidence, scalp comfort and scalp health improved over time, irrespective of product type. Fourth, there was no change in measures of self‐esteem for either shampoo. Subsequently, the mediation analysis revealed that across both studies, ADS treatment improved symptoms, emotional wellbeing, and functioning ratings on the Scalpdex questionnaire. Importantly, it also found that improvements in physical symptoms of dandruff improved behavioural functioning indirectly, via its mediating effect on scalp‐related emotional wellbeing.

**Conclusions:**

This study demonstrated that reducing dandruff's physical symptoms directly enhanced emotional well‐being related to scalp and hair, and indirectly improved overall daily functioning. These findings suggest that addressing dandruff symptoms not only increases physical comfort but also positively impacts individuals' mental and emotional health, and their ability to function in their daily lives.

## Graphical abstract

This study demonstrated that reducing dandruff's physical symptoms directly enhanced scalp and hair‐related emotional well‐being, and indirectly improved overall daily functioning. Addressing dandruff symptoms not only increases physical comfort but also positively impacts individuals' mental and emotional health, and their ability to function in their daily lives.

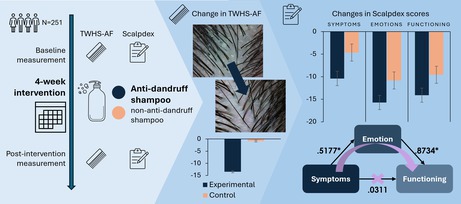

## Résumé

**Objectif:**

Les pellicules sont une affection fréquente du cuir chevelu caractérisée par la perte chronique de gros amas de cellules épidermiques. En plus des symptômes physiques négatifs, les pellicules auraient un impact préjudiciable sur la santé mentale des patients. L'objectif de cette étude était de déterminer si les bénéfices cliniques de l'utilisation d'un shampooing antipelliculaire (SA) permettent d'améliorer le bien‐être psychologique des patients ayant des pellicules. En outre, cette étude visait à déterminer si cette amélioration se manifesterait par des changements dans l'estime de soi et dans les comportements liés à la confiance en soi.

**Méthodes:**

Une intervention de réduction des pellicules de 4 semaines a été mise en œuvre à l'aide d'une formulation de SA à base de piroctone olamine. Les patients du groupe témoin ont continué à utiliser leur shampooing non antipelliculaire. Des indicateurs d'auto‐évaluation, notamment l'échelle Scalpdex, l'échelle de l'estime de soi State (State self‐esteem scale) et les questionnaires sur la perception des caractéristiques des cheveux et du cuir chevelu, ont été utilisés pour mesurer les changements dans les perceptions sur la santé et le bien‐être psychologique liés aux cheveux et au cuir chevelu. Suite à cette analyse, l'ensemble de données a ensuite été combiné avec des données internes non publiées d'une étude interventionnelle similaire utilisant une formulation de SA à base de pyrithione de zinc. Une analyse de médiation a été réalisée sur les données combinées pour examiner la relation entre les symptômes des pellicules, les émotions et le fonctionnement comportemental global.

**Résultats:**

Tout d'abord, le SA a réduit efficacement les symptômes cliniques des pellicules. Deuxièmement, les scores Scalpdex indiquaient que l'utilisation de SA, par rapport à l'absence d'utilisation de SA, diminuait les effets indésirables des pellicules sur la perception des symptômes et la détresse émotionnelle. Troisièmement, l'impact des pellicules sur le fonctionnement comportemental a diminué au fil du temps, et les mesures de la confiance, du confort du cuir chevelu et de la santé du cuir chevelu se sont améliorées au fil du temps, indépendamment du produit utilisé. Quatrièmement, il n'y a pas eu de changement dans les mesures de l'estime de soi pour l'un ou l'autre shampooing. Ensuite, l'analyse de médiation a révélé que, dans les deux études, le traitement par SA améliorait les symptômes, le bien‐être émotionnel et les scores de fonctionnement sur le questionnaire Scalpdex. Il est. important de noter que les améliorations des symptômes physiques des pellicules ont indirectement amélioré le fonctionnement comportemental, grâce à leur effet médiateur sur le bien‐être émotionnel lié au cuir chevelu.

**Conclusions:**

Cette étude a démontré que la réduction des symptômes physiques des pellicules améliorait directement le bien‐être émotionnel lié au cuir chevelu et aux cheveux, et améliorait indirectement le fonctionnement quotidien global. Ces résultats suggèrent que la prise en charge des symptômes des pellicules augmente non seulement le confort physique, mais a également un impact positif sur la santé mentale et émotionnelle des patients, ainsi que sur leur capacité à fonctionner dans la vie quotidienne.

## INTRODUCTION

Dandruff is a common issue affecting the hair and scalp, with approximately half of the global population experiencing dandruff at some point in their lives [1, 2, 3, 4]. A chronic condition caused by shedding of large clumps scalp of epidermal cells, dandruff is typically characterized by white or grey flakes of dead skin visible on the scalp and in the hair [5]. It is often accompanied with scalp itchiness and dryness, and can impact hair health causing brittleness, dullness, and hair loss [6]. In addition to physical symptoms, skin disorders similar to dandruff, such as psoriasis and eczema, have been shown to significantly impact mental and emotional wellbeing by causing psychological distress, particularly in social situations [3, 7, 8].

Visible bodily conditions that are considered socially undesirable have been found to have a significant negative impact on important psychosocial characteristics such as confidence and self‐esteem [9, 10, 11]. Sociometer Theory suggests that self‐esteem serves as an evolutionary adaptation that helps individuals assess which characteristics are socially valued and acceptable, enabling them to avoid social exclusion and rejection from social groups [12]. As self‐esteem is thought to reflect an individual's self‐perception in relation to others, it can fluctuate depending on how valued and accepted they feel within their social group [12, 13]. Dandruff can result in itching behaviours and the presence of undesirable scalp skin flakes, which can be seen by both the affected individual and others in close proximity. As a result, individuals with dandruff may be perceived as untidy, unhygienic, and unattractive [3]. Consequently, knowing about and noticing one's dandruff is likely to have a negative impact on psychosocial wellbeing, manifesting as reduced confidence and lowered self‐esteem.

However, despite the prevalence of dandruff as a chronic skin condition, the scientific literature exploring the impact of dandruff on psychosocial wellbeing is still in its infancy. Most studies have focused on more severe and chronic skin conditions such as seborrhoeic dermatitis, eczema, and psoriasis, which can affect areas beyond the scalp, including the face, limbs, and chest [14]. Dandruff is also often conflated with these other conditions as they are considered to exist on a spectrum [14]. As a result, several studies examining the psychosocial symptoms of dandruff have also included individuals with seborrhoeic eczema and seborrhoeic psoriasis [15]. However, within this body of literature, there is consistent evidence indicating that the more severe presentation of seborrhoeic dermatitis symptoms is associated with higher stress levels, lower quality of life and increased experiences of anxiety and depression symptoms, including clinically diagnosed depression [15, 16, 17]. From this, it is plausible that dandruff will cause a similar detrimental impact to individual's emotional and psychological wellbeing.

More recently, Godbehere et al. [13] examined psychosocial functioning in individuals with dandruff. They found that the severity of symptoms was linked to a greater negative impact on everyday functioning and emotional wellbeing. Interestingly, although individuals with dandruff scored lower on the Scalpdex quality of life questionnaire [18], they did not report any reduction in self‐perceived confidence, attractiveness, or self‐esteem compared to individuals without dandruff. Moreover, during preparation for and undergoing a mock interview, impartial third‐party observers did not rate female dandruff participants differently from female non‐dandruff participants in terms of their confidence or attractiveness. Interestingly, male participants with dandruff were rated as less confident than those without dandruff. These findings suggest several important points. First, this is preliminary evidence of a link between dandruff and psychosocial functioning, indicating that dandruff can have an impact on an individual's overall wellbeing beyond physical symptoms alone. The impressions on third‐party observers suggest that the presence of dandruff can visibly change behaviours. Second, there is a potential gender disparity in the influence of dandruff. This may be due to a difference of the psychological resonance of dandruff on men compared to women, or may be due to broader use of cosmetic interventions in women which could reduce, mask or mitigate the effect on body language perception in these scenarios. Further research would be needed to fully elaborate upon these findings. Lastly, the findings indicate a dissociation between physical symptomology, internal emotional processing, and externally perceived behaviour and functioning. In other words, individuals with dandruff may not explicitly consciously experience lower self‐confidence, but the detrimental impact may manifest subconsciously in behaviours and body language. These insights highlight the complex relationship between dandruff, psychosocial factors, and external perceptions, and warrant further investigation to gain a comprehensive understanding of these dynamics.

This paper details an experiment we conducted to explore the causal relationship between dandruff symptoms, emotional wellbeing, and behavioural functioning. A mixed‐gender sample underwent a 4‐week intervention: an anti‐dandruff shampoo (ADS) usage was implemented to reduce physical symptomology for comparison with a matched control group using their regular non‐anti‐dandruff shampoo (NADS). Participants completed self‐report measures of psychological wellbeing before and after the intervention. We predicted that participants in the ADS group would experience clinical benefits to their scalp condition, and this reduction in symptom severity would directly result in improved scores on self‐evaluation wellbeing measures. Subsequently, the current study was combined with unpublished data from a similar paradigm utilizing a Zinc Pyrithione ADS formulation and an all‐male sample. A mediation analysis was conducted to investigate the causal relationship between changes in the Scalpdex subscales of symptoms, psychological consequences, and differences in functioning using a larger dataset. We hypothesized that the emotional benefits of dandruff removal would mediate any effect on observable and implicit behaviour, body language and functioning changes.

## EXPERIMENT 1

### Methods

#### Participants

Participants were recruited from the Xi'an area in China, and gave their full informed consent to procedures which were screened by the Independent Ethics Committee of the Shanghai Clinical Research Centre (SCRC) which abided by the Declaration of Helsinki. To qualify for inclusion, participants needed to be aged 18–30 years old, be a shampoo user, computer literature, have finished school, self‐identify as a dandruff sufferer, and were clinically approved by an accredited scalp assessor to have clinical dandruff based on having a Total Weighted Head Score Adherent Flakes (TWHS‐AF) of 28 or greater. Participants were excluded if they were a user of anti‐dandruff products, or suffered from any severe skin condition. Participants were remunerated for their time and effort.

A total of 340 participants were recruited, of which 261 participants passed the TWHS‐AF clinical scalp assessment and so took part in the experiment. Participants were pseudo randomly allocated to the experimental group or the control group, with an equal split of gender. 249 participants (129 women) returned after 4 weeks for the second visit to complete the experiment. In the experimental group (*N* = 125, 65 women), participants were given a Piroctone olamine ADS to use at home for 4 weeks. Control group participants (*N* = 124, 64 women) used their own NADS for 4 weeks.

#### Procedure

##### Clinical scalp assessment

Dandruff prevalence was assessed according to scalp flaking using the TWHS‐AF method, which is a well‐defined approach to assess flake severity [2, 19, 20, 21]. The method involves an expert clinical assessor examining the scalp under standardized lighting conditions and grading the dryness, blemishes and erythema severity on a number of scales. The total area covered in 10% increments is graded either a completely healthy scalp (0) or one of five different levels of severity: from minimal dry powdery flakes (A) to the most severe grade (E) for substantially crusting dandruff and blemishes. The estimate of the surface area for each grade is multiplied by the weighting score allocated to that severity grade (0 for perfect scalp; 5 for large crusting flakes). These scores are then added together, providing the weighted head score, and this is multiplied by a factor appropriate to the assessment type, providing the final TWHS‐AF [22]. A clinical scalp assessment was conducted using the TWHS‐AF method at Visit 1 and Visit 2 to assess the clinical efficacy of the intervention product on scalp condition.

##### Self‐evaluation metrics

Participants were asked to complete a series of explicit self‐evaluation measures according to how they currently felt at Visit 1 (prior to intervention as a baseline measure) and again at Visit 2 (following 4 weeks of experimental or control intervention). The self‐report measures comprised: self‐perceived attractiveness and confidence, self‐perceived hair and scalp characteristics ratings, the Scalpdex index [18] and the State Self‐Esteem Scale (SSES) [23].

###### Confidence and comfort

Participants were asked five questions regarding how they felt about their scalp comfort, holistic scalp health, scalp confidence, hair confidence and self‐confidence. Participants answered using a line scale −100 to +100.

###### Scalpdex

The Scalpdex is a clinical quality‐of‐life instrument developed to quantify the impact of scalp pathology, specifically scalp dermatitis, on patients' quality of life [18]. The scale comprises 23 questions around 3 subscales: functioning (How do your scalp issues affect your everyday ability to function?), symptoms (such as itchiness, flakiness, tightness of skin) and emotions (increased stress, changes in mood). Following procedures outlined by Chen et al. [18], greater Scalpdex scores reported here reflect a greater negative impact from dandruff on emotions, symptoms or functioning.

###### State Self‐Esteem Scale (SSES)

The SSES is a 20‐item questionnaire that measures current self‐esteem [23]. Participants provided ratings on a 1 (do not at all agree) to 5 (completely agree) scale, and averaged ratings were used to generate an overall self‐esteem score so that higher SSES values indicated higher self‐esteem overall.

#### Statistical analysis

A series of mixed measures ANOVAs were conducted exploring differences in the TWHS‐AF and self‐evaluation metrics between the experimental and control groups (between‐group independent variable) between Visit 1 and Visit 2 (within‐group independent variable). Greenhouse Geiser corrections were utilized whenever the sphericity assumption was violated, and significance threshold of *p* < 0.05 was employed.

### Results

#### Clinical scalp assessment

A 2 × 2 mixed ANOVA revealed there was a main effect of visit (*F*(1, 247) = 573.97, *p* < 0.001, *ηp* ^2^ = 0.699), of treatment (*F*(1, 247) = 210.36, *p* < 0.001, *ηp* ^2^ = 0.460), and a significant interaction effect (*F*(1, 247) = 447.04, *p* < 0.001, *ηp* ^2^ = 0.644). There was a statistically significant reduction in dandruff, as measured by TWHS‐AF, between Visit 1 and Visit 2. Post hoc paired sampled *t*‐tests revealed that the experimental group showed TWHS‐AF scores reduced by 13.44 between Visit 1 and Visit 2 (*t*(124) = 29.97, *p* < 0.001), confirming the clinical efficacy of the ADS intervention. The control group also showed a small but significant reduction in dandruff severity of 0.84 between Visit 1 and Visit 2 (*t*(123) = 2.139, *p* = 0.034). However, this reduction did not fall below the threshold for clinical dandruff (>28 TWHS‐AF), and so was not clinically significant. The change in baseline between the two groups was significantly different (*t*(247) = 21.15, *p* = 0.001).

#### Scalpdex

**FIGURE 1:**
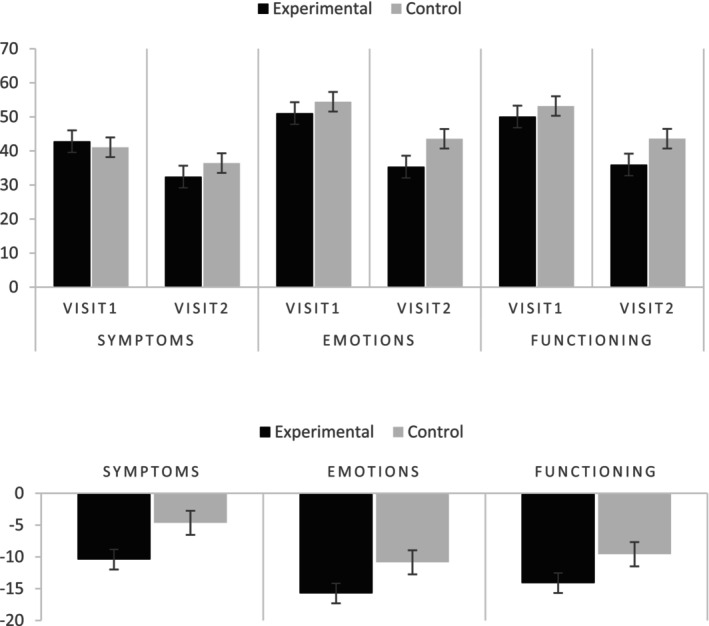
Top: Mean Scalpdex subscale scores for experimental and control group for Visit 1 and Visit 2. Bottom: Post‐intervention changes from baseline in Scalpdex constructs for experimental and control groups. Error bars ± SE.

Scalpdex questionnaire scores were averaged for each of the three constructs at the participant level so that a higher score reflects a greater negative impact from dandruff (following procedures outlined by Chen et al., 2003). 2 × 2 (group × visit) mixed ANOVAs were conducted for each construct. All three constructs show a main effect of visit (Symptoms: *F*(1, 247) = 41.31, *p* < 0.001, *ηp* ^2^ = 0.143; Emotion: *F*(1, 247) = 143.26, *p* < 0.001, *ηp* ^2^ = 0.367; Function: *F*(1, 247) = 77.84, *p* < 0.001, *ηp* ^2^ = 0.240), where in all cases scores were lower post‐intervention. Figure 1 shows mean score values for each condition (top) and the differences in average scores for the three constructs of the Scalpdex questionnaire (bottom).

For Symptoms, there was no main effect of treatment group (*p* > 0.05) but a significant interaction effect between group and visit (*F*(1, 247) = 6.07, *p =* 0.014, *ηp* ^2^ = 0.024), where the difference between the visits was much greater for the experimental group (−10.40) compared to the control group (−4.64).

For Emotion, the control group had higher average scores than the experimental group (*F*(1, 247) = 7.71 *p =* 0.006, *ηp* ^2^ = 0.030; experimental: 43.18, control: 49.00); and a significant interaction effect (*F*(1, 247) = 4.82, *p =* 0.029, *ηp* ^2^ = 0.019) showed that score reductions between the two visits were greater in the experimental group (−15.73) compared to the control group (−10.85).

Finally, for Function, again the control group had higher average scores than the experimental group (*F*(1, 247) = 5.28, *p =* 0.022, *ηp* ^2^ = 0.021; experimental: 43.00, control: 48.39), but no significant interaction effect was found (*p* > 0.05).

#### Self‐evaluation procedures

A series of 2 × 2 mixed ANOVAs (visit × group) explored participant ratings for scalp comfort, holistic scalp health, scalp confidence, hair confidence and self‐confidence, and also SSES scores on dimensions of appearance, performance, and social self‐esteem. Significant main effects for individual self‐evaluation constructs can be seen in Table 1. There were no main or interactions effects of group (ADS/NADS) or of Visit (1/2) found for SSES scores (all *ps* >0.05).

**TABLE 1:** Results of mixed ANOVAs (visit × group) for self‐evaluation procedures.

Self‐evaluation construct	Main effect of time	Main effect of condition	Interaction
Scalp comfort	*F*(1, 247) = 53.27, *p* < 0.001, *ηp* ^2^ = 0.177	F(1, 247) = 5.01, *p* < 0.026, *ηp* ^2^ = 0.020	–
Holistic scalp health	*F*(1, 247) = 48.95, *p* < 0.001, *ηp* ^2^ = 0.165	–	–
Scalp confidence	*F*(1, 247) = 30.99, *p* < 0.001, *ηp* ^2^ = 0.111	–	–
Hair confidence	*F*(1, 247) = 37.08, *p* < 0.001, *ηp* ^2^ = 0.131	–	–
Self‐confidence	*F*(1, 247) = 24.29, *p* < 0.001, *ηp* ^2^ = 0.090	–	–

### Conclusion

The key aim of this experiment was to determine whether the clinical benefits of using an ADS will result in improved quality of life and increased confidence in dandruff sufferers. To address this question, we measured quality of life through a range of self‐evaluation metrics. Our findings can be summarized as follows. First, ADS product use was found to lead to clinical improvements in scalp condition. Second, Scalpdex scores showed ADS product use, relative to NADS product use, reduced the negative impact from dandruff on symptom and emotions perceptions, with the impact on functioning reducing over time regardless of type of product use. Third, measures of confidence and scalp comfort and health improved over time and were not dependent on type of product used. Fourth, global self‐report measures of self‐esteem were not affected by product use.

## MEDIATION ANALYSIS

### Methods

**FIGURE 2:**
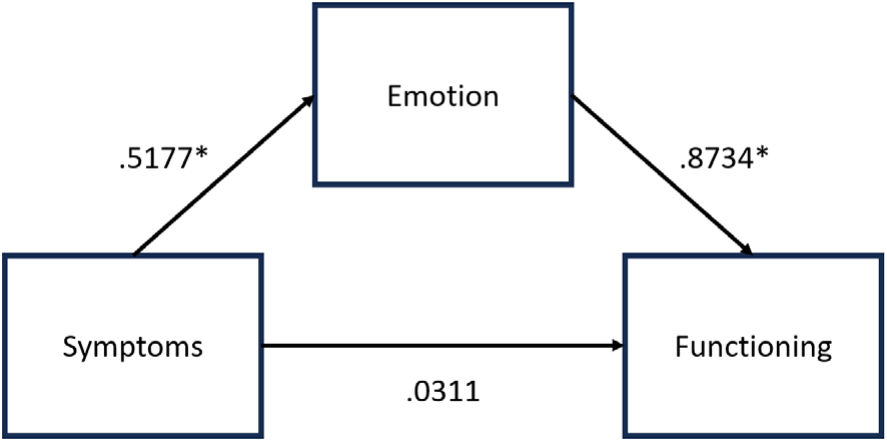
Mediation analysis model between symptoms (IV) function (DV) and emotions (MV). *Indicates significance at *p* < 0.001.

Scalpdex questionnaire data from Experiment 1 was combined with data from a comparable internal study (Experiment 2), where a group of healthy males (47 experimental and 42 control, mean (±SD) age of 24 (±3.2) years) were given a Zinc Pyrithione ADS or used their own NADS to use at home for 4 weeks, and completed the Scalpdex index prior and post intervention. The total combined cohort for the mediation analysis was *N* = 171 in the experimental group and *N* = 166 in the control group. A multivariate ANOVA was conducted with time (Visit 1/2) as the within‐subject variable and treatment as the between subject variable, and the three Scalpdex subscales as outcome variables. Next, change scores were calculated between Visit 1 and 2 for all three Scalpdex subscales, and a Pearson's correlation analysis was conducted to understand the relationship between the change scores for each symptom cluster. A simple mediation analysis was then conducted using a bootstrapping approach with 5000 permutations performed by the PROCESS SPSS macro [24, 25, 26]. Figure 2 depicts the mediation analysis model.

### Results

The multivariate ANOVA found critical significant interaction between time and treatment [*F*(2,336) = 4.301, *p* = 0.014]. Follow‐up univariate tests confirmed an interaction for treatment type and visit for all three subscales of the Scalpdex (see Table 2). These analyses demonstrate a greater reduction across all three symptom clusters after experimental treatment compared to the control condition. As can be seen in Table 3, change scores on all three subscales correlated significantly with medium to high effect sizes.

**TABLE 2:** Univariate tests confirmed an interaction for treatment type and visit for all subscales of the Scalpdex pooled Experiment 1 and Experiment 2 unpublished data.

Treatment	Visit 1 – Visit 2	df	*t*	*p*
Experimental	Symptoms	170	5.641	<0.001
	Emotions	170	9.610	<0.001
	Functions	170	7.714	<0.001
Control	Symptoms	165	2.087	0.038
	Emotions	165	6.109	<0.001
	Functions	165	3.967	<0.001

**TABLE 3:** Pearson's correlation analysis between change scores (between Visit 1 and Visit 2) for each of the Scalpdex subscales of symptoms, emotions, and functioning.

	Symptoms	Emotions	Functions
Symptoms	–		
Emotion	0.544**	–	
Functioning	0.427**	0.750**	–

** *p* < 0.01.

The mediation analysis revealed that symptoms were positively associated with emotions (*a* = 0.5177, SE = 0.0435, *p* < 0.001), and emotions were positively associated with functioning (*b* = 0.8734, SE = 0.0511, *p* < 0.001); however, there was no significant direct association between symptoms and functioning (*c*′ = 0.0311, SE = 0.0486, *p* = 0.523). Bootstrapping revealed that scores on the emotion subscale mediated the relationship between symptom scores and functioning scores: *ab* = 0.4522, 95% CI: 0.3373–0.5807, *p* < 0.001.

### Conclusion

Pooled data across Experiment 1 and 2 enabled the investigation into how improvements in scalp‐related quality of life, as measured on the Scalpdex subscales, relate to each other. First, across both studies, ADS treatment, compared to the control condition, improved symptoms, emotional wellbeing, and functioning ratings on the Scalpdex. Second, improvements in physical symptoms improved function indirectly via its mediating effect on scalp‐related emotional wellbeing.

## DISCUSSION

The current study explored the relationship between physical symptomology and emotional wellbeing experiences in dandruff sufferers, and how the objective efficacy of anti‐dandruff products may be differentiated from their subjectively perceived benefits. Experiment 1 demonstrated that a clinically significant reduction of dandruff severity, following 4 weeks of using ADS, led to improvements in self‐reported scalp‐related quality of life. By combining the Experiment 1 cohort with a second dataset, we found a strong indirect effect of symptom reduction on daily functioning, which was mediated by scalp‐ and hair‐related emotional processing. In addition to the Scalpdex analyses, we found no impact of improvement in dandruff symptomology on self‐reported self‐esteem or self‐confidence. Taken together, the results suggest that improvements in physical symptoms have a differentiated impact on the processing of trait and state emotions.

In Experiment 1, ADS users showed a greater reduction in clinical scalp condition compared to matched controls, and in the Scalpdex subscales of scalp‐ and hair‐related symptoms and emotional issues [18]. The findings of this study align with existing literature on the psychosocial consequences of visible and chronic skin conditions, such as vitiligo [27], eczema, acne, psoriasis [28], and seborrhoeic dermatitis [29]. Consistent with previous research by Godbehere et al. [13], the results provide converging evidence of the negative psychological impact of dandruff.

To further examine the directionality of the relationship between the physical and psychological impact of dandruff, a mediation analysis was conducted using combined data from Experiment 1 and 2. The analysis revealed that improvements in scalp‐related emotions mediated the relationship between dandruff symptom severity and daily functioning. This suggests that the negative psychological impact of dandruff, such as feelings of depression, self‐consciousness, and alienation, can result in certain behaviours being affected. These behaviours may include making clothing and styling choices based on the presence of dandruff, as well as avoiding grooming activities such as going to the hairdresser. This finding is supported by research indicating that emotion regulation is associated with quality of life in people suffering from chronic skin conditions [30, 31, 32]. In individuals with clinical skin conditions, the regulation of negative emotions improves overall quality of life, such as better wellbeing and lower levels of distress [30]. Conversely, emotional dysregulation and psychological distress can worsen dermatological conditions, subsequently diminishing quality of life [33]. Analogously, our findings suggest that the use of ADS improves quality of life through symptom reduction, which in turn enhances scalp and hair‐related emotions, which in turn improves daily functioning.

In line with the findings from Godbehere et al. [13], we did not find a between‐group difference in participant evaluations of self‐esteem or self‐confidence. Godbehere and colleagues proposed that improvements in self‐worth may not be consciously perceived, and as such, any changes in self‐esteem may not be established or observable within a short time period. Self‐worth is a complex psychological and emotional construct that may not be immediately influenced solely by the resolution of physical symptoms, such as a reduction in dandruff in this case. Therefore, any implicit change in self‐confidence may take consistent intervention to undergo restructuring before being manifested in observable behaviours, compared to the more transient, explicit, and emotion‐related changes captured in the Scalpdex questionnaire. Furthermore, there may be a different psychological impact of the absence of dandruff (vs. presence previously, or vice versa) compared to a change in severity of dandruff. Future studies could further investigate this by implementing a more prolonged intervention to establish the impact of long‐term sustained changes in dandruff severity on self‐esteem and self‐confidence.

There is a substantial body of research that provides evidence of psychological distress triggering the occurrence of clinical skin disorders and exacerbating their frequency and severity. For example, in a large‐scale longitudinal study of patients with seborrhoeic dermatitis, 76% of the cohort (*N* = 2159) reported that psychological factors triggered an outbreak [34]. From this evidence, a reciprocal relationship between the occurrence of dandruff and an individual's psychological wellbeing is plausible, but it is yet to be systematically established. Since the present study focused on participants already experiencing severe dandruff, it was beyond its scope to investigate the influence of anxiety, depression, fatigue or stress in triggering or worsening dandruff symptoms. Future research could explore and confirm the mechanisms underlying the reciprocal relationship between negative emotions and the experience of dandruff symptoms.

Taken together, the reduction of physical dandruff symptoms through ADS use reduces negative scalp and hair‐related emotions, leading to better daily functioning. This connection between symptom reduction and emotions is vital for dandruff sufferers, likely because of the strong links between emotions, stress, psychological health, and skin inflammation. These studies highlight the significance of emotions in the treatment and functional outcomes of individuals with dandruff. Going forward, it is crucial to prioritize scalp and hair‐related emotional wellbeing in anti‐dandruff product development to improve long‐term, sustainable results for individuals with chronic dermatological conditions.

## AUTHOR CONTRIBUTIONS

AG, LC, TJ & MS contributed to the development of the experimental design and planning of this work and conducted the data acquisition. ANF and HR conducted statistical analyses for experiments 1 and 2, TG conducted the mediation analysis. ANF and HR produced the final written manuscript, which was overseen by TG. All authors reviewed and provided feedback on the final manuscript.

## FUNDING INFORMATION

The work was funded by Unilever U.K. Central Resources Limited.

## CONFLICT OF INTEREST STATEMENT

ANF, MS, TJ, LC and TG work for Unilever.

## References

[1] Elewski BE . Clinical diagnosis of common scalp disorders. J Investig Dermatol Symp Proc. 2005;10(3):190–193. 10.1111/j.1087-0024.2005.10103.x 16382661

[2] Harding CR , Moore AE , Rogers SJ , Meldrum H , Scott AE , McGlone FP . Dandruff: a condition characterized by decreased levels of intercellular lipids in scalp stratum corneum and impaired barrier function. Arch Dermatol Res. 2002a;294:221–230. 12115025 10.1007/s00403-002-0323-1

[3] Manuel F , Ranganathan S . A new postulate on two stages of dandruff: a clinical perspective. Int J Trichol. 2011;3(1):3–6. 10.4103/0974-7753.82117 PMC3129121 21769228

[4] Wilson M , Wilson PJ . Close Encounters of the Microbial Kind; 2021. p. 451–98. Cham: Springer.

[5] Zoya M , Bhikhu M , Gaurav S . Anti‐dandruff activity of synthetic and herbal shampoos on dandruff causing isolate: Malassezia. Int J Appl Res. 2016;2:80–85.

[6] Schwartz JR , Messenger AG , Tosti A , Todd G , Hordinsky M , Hay RJ , et al. A comprehensive pathophysiology of dandruff and seborrheic dermatitis–towards a more precise definition of scalp health. Acta Derm Venereol. 2013;93(2):131–137. 22875203 10.2340/00015555-1382

[7] Evers AWM , Lu Y , Duller P , Van Der Valk PGM , Kraaimaat FW , Van De Kerkhof PCM . Common burden of chronic skin diseases? Contributors to psychological distress in adults with psoriasis and atopic dermatitis. Brit J Dermatol. 2005;152(6):1275–1281. 15948993 10.1111/j.1365-2133.2005.06565.x

[8] Sibbald RG , Ayello EA . Psychophysiology: connecting skin, wounds, aging, and depression. Advances in Skin & Wound Care. 2021;34:399. 34260416 10.1097/01.ASW.0000758600.40793.89

[9] Dalgard F , Gieler U , Holm JØ , Bjertness E , Hauser S . Self‐esteem and body satisfaction among late adolescents with acne: results from a population survey. J Am Acad Dermatol. 2008;59(5):746–751. 19119094 10.1016/j.jaad.2008.07.013

[10] Erturan İ , Aktepe E , Balcı DD , Yıldırım M , Sönmez Y , Ceyhan AM . Evaluation of self‐esteem and dermatological quality of life in adolescents with atopic dermatitis. Turkderm. 2013;47:39–44.

[11] Kokandi A . Evaluation of acne quality of life and clinical severity in acne female adults. Dermatol Res Pract. 2010;2010(1):410809. 20706683 10.1155/2010/410809 PMC2913789

[12] Leary MR . Sociometer theory. Theories Soc Psychol. 2012:2;141–159.

[13] Godbehere A , McDonald L , Baines F , Sutherland CAM , Andrews TJ . A dissociation in judgements of confidence in people with dandruff based on self‐reports compared to reports from other observers. Int J Cosmet Sci. 2017;39(4):457–464. 28375586 10.1111/ics.12400

[14] Ranganathan S , Mukhopadhyay T . Dandruff: the most commercially exploited skin disease. Indian J Dermatol. 2010;55(2):130–134. 20606879 10.4103/0019-5154.62734 PMC2887514

[15] Misery L , Touboul S , Vincot C , Dutray S , Rolland‐Jacob G , Consoli SG , et al. Stress and seborrheic dermatitis. Ann Dermatol Venereol. 2007;134(11):833–837. 18033062 10.1016/s0151-9638(07)92826-4

[16] Aksoy M , Özkorumak E , Bahadır S , Yaylı S , Aksu Arıca D . Quality of life, anxiety and depression levels in patients with seborrheic dermatitis. 2012.

[17] Maietta G , Fornaro P , Rongioletti F , Rebora A . Patients with mood depression have a high prevalence of seborrhoeic dermatitis. Acta Derm Venereol. 1990;70(5):432–434. 1980980

[18] Chen SC , Yeung J , Chren M‐M . Scalpdex: a quality‐of‐life instrument for scalp dermatitis. Arch Dermatol. 2002;138(6):803–807. 12056963 10.1001/archderm.138.6.803

[19] Collins LZ , Baines FL , Matheson JR , Turner GA , Diao Y , Li Y , et al. Sex‐related differences in response to zinc pyrithione shampoo vs. non‐anti‐dandruff shampoo. Int J Cosmet Sci. 2018;40(6):583–588. 30447108 10.1111/ics.12501

[20] Harding CR , Matheson JR , Hoptroff M , Jones DA , Luo Y , Baines FL , et al. A high glycerol‐containing leave‐on scalp care treatment to improve dandruff. Skinmed. 2014;12(3):155–161. 25134312

[21] Turner GA , Matheson JR , Li G , Fei X , Zhu D , Baines FL . Enhanced efficacy and sensory properties of an anti‐dandruff shampoo containing zinc pyrithione and climbazole. Int J Cosmet Sci. 2013;35(1):78–83. 22970742 10.1111/ics.12007

[22] Diao Y , Matheson JR , Pi Y , Baines FL , Zhang S , Li Y . Comparison of whole‐head and split‐head design for the clinical evaluation of anti‐dandruff shampoo efficacy. Int J Cosmet Sci. 2021;43(5):510–517. 34058011 10.1111/ics.12718 PMC9290732

[23] Heatherton TF , Polivy J . Development and validation of a scale for measuring state self‐esteem. J Pers Soc Psychol. 1991;60(6):895–910.

[24] Hayes AF . PROCESS: a versatile computational tool for observed variable mediation, moderation, and conditional process modeling. Lawrence, KS, USA: University of Kansas; 2012.

[25] Hayes AF . An index and test of linear moderated mediation. Multivar Behav Res. 2015;50(1):1–22. 10.1080/00273171.2014.962683 26609740

[26] Preacher KJ , Hayes AF . SPSS and SAS procedures for estimating indirect effects in simple mediation models. Behav Res Methods Instrum Comput. 2004;36:717–731. 15641418 10.3758/bf03206553

[27] Porter J , Beuf AH , Nordlund JJ , Lerner AB . Psychological reaction to chronic skin disorders: a study of patients with vitiligo. Gen Hosp Psychiatry. 1979;1(1):73–77. 499777 10.1016/0163-8343(79)90081-1

[28] Magin P , Heading G , Adams J , Pond D . Sex and the skin: a qualitative study of patients with acne, psoriasis and atopic eczema. Psychol Health Med. 2010;15(4):454–462. 20677083 10.1080/13548506.2010.484463

[29] Borda LJ , Wikramanayake TC . Seborrheic dermatitis and dandruff: a comprehensive review. J Clin Investig Dermatol. 2015;3(2):158–77. 10.13188/2373-1044.1000019 PMC4852869 27148560

[30] Iani L , Quinto RM , Porcelli P , Angeramo A‐R , Schiralli A , Abeni D . Positive psychological factors are associated with better spiritual well‐being and lower distress in individuals with skin diseases. Front Psychol. 2020;11:552764. 33123038 10.3389/fpsyg.2020.552764 PMC7573544

[31] Innamorati M , Quinto RM , Lester D , Iani L , Graceffa D , Bonifati C . Cognitive impairment in patients with psoriasis: a matched case‐control study. J Psychosom Res. 2018;105:99–105. 29332640 10.1016/j.jpsychores.2017.12.011

[32] Quinto RM , Sampogna F , Fania L , Ciccone D , Fusari R , Mastroeni S , et al. Alexithymia, psychological distress, and social impairment in patients with hidradenitis suppurativa. Dermatology. 2021;237(1):103–110. 31743903 10.1159/000503319

[33] Ciuluvica C , Fulcheri M , Amerio P . Expressive suppression and negative affect, pathways of emotional dysregulation in psoriasis patients. Front Psychol. 2019;10:1907. 31496974 10.3389/fpsyg.2019.01907 PMC6712996

[34] Peyri J , Lleonart M . Clinical and therapeutic profile and quality of life of patients with seborrheic dermatitis. Actas Dermo‐Sifiliográficas. 2007;98(7):476–482. 17669302

